# A Novel In-Circuit Impedance Modeling Method and Variation Characteristics Analysis for SMPS

**DOI:** 10.3390/mi17020232

**Published:** 2026-02-11

**Authors:** Jun Zhan, Ziliang Zhang, Rongxuan Zhang, Chunying Gong, Jie Chen

**Affiliations:** College of Automation Engineering, School of Automation, Nanjing University of Aeronautics and Astronautics (NUAA), Nanjing 211106, China; zhanjun@nuaa.edu.cn (J.Z.); zhangziliang@nuaa.edu.cn (Z.Z.); rxzhang01@nuaa.edu.cn (R.Z.); zjnjgcy@nuaa.edu.cn (C.G.)

**Keywords:** in-circuit impedance, inductive coupling method, switched-mode power supply (SMPS), EMI filter

## Abstract

The precise in-circuit impedance extraction in a switched-mode power supply (SMPS) is essential for the optimal design of electromagnetic interference (EMI) filters. The design of EMI filter parameters based on in-circuit impedance has already been widely investigated in the literature, but the variation characteristics of the in-circuit impedance for an SMPS is still a research gap and needs further study. In this article, based on the concept of the inductive coupling approach, a novel method for in-circuit impedance modeling is proposed. Subsequently, an accurate in-circuit impedance modeling is derived, which indicates that the in-circuit impedance for the SMPS is related to the external impedance, the modal impedance under different switching modes, and the proportion of each switching mode. Based on the derived model, the variation characteristics of the in-circuit impedance are revealed, which can provide valuable guidance for the design of EMI suppression measures. Finally, the simulation results show good agreement with the calculated results. Experimental verification further indicates that the model accurately characterizes the impedance of the switching power supply across the range of 10 kHz to 30 MHz, with amplitude deviation within 3 dB and phase deviation below 6 degrees. This work provides a quantitative foundation for designing electromagnetic interference suppression strategies, enabling more precise filter optimization over a broad frequency range.

## 1. Introduction

EMI filters are the most commonly used components to effectively suppress EMI [1,2,3,4]. In-circuit impedance is one of the key parameters for EMI filter design and has a significant impact on its filtering performance. Extracting the in-circuit impedance is helpful for the targeted selection of EMI filter type, topology, and component parameters, enables accurate prediction of the insertion loss of EMI filters, and provides a reliable basis for the precise forward design of EMI filters [5,6].

There are three existing approaches for measuring the in-circuit impedance of an SMPS: the voltage-current approach [7,8], the capacitance coupling approach [9,10], and the inductive coupling approach. The inductive coupling approach, which has no direct electrical interconnection between the measurement equipment and SMPS, has proven to be more secure and convenient, and is widely used. In Ref. [11], the inductive coupling approach was first proposed and applied for extracting the in-circuit impedance of an SMPS for designing EMI filters. A signal generator injects a small signal into the SMPS using an injection inductive probe (IIP), and a spectrum analyzer receives the response signal using a receiving inductive probe (RIP). In Ref. [12], a vector network analyzer (VNA) was used for injecting and receiving signals into an SMPS. The S-parameters measured by the VNA can be used to calculate the in-circuit impedance. In the actual measurement process, two inductive probes used in this approach may be very close to each other, and the probe-to-probe coupling between inductive probes cannot be ignored. In Ref. [13], an open-short-load calibration technique was proposed to eliminate potential errors contributed by the probe-to-probe coupling. As the switching frequency of the SMPS increases, the power noise of the SMPS becomes nonnegligible relative to the VNA-injected signals. An in-circuit impedance measurement model and an accurate measurement technique were proposed in Ref. [14] to reduce the measurement error of the in-circuit impedance caused by stationary power noise of the SMPS. The authors of Ref. [15] proposed a two-port, inductively coupled in-circuit impedance method, which can provide accurate measurement of admittance parameters, including mutual terms, both for active and passive two-port devices. In Ref. [16], time-variant in-circuit impedance monitoring based on the inductive coupling approach was proposed, which can measure the real-time in-circuit impedance of an SMPS. On this basis, Ref. [17] proposed a single-probe setup based on the inductive coupling approach, which can perform multifrequency simultaneous measurement. The experimental results of Refs. [16,17] reveal that the in-circuit impedance varies notably when an SMPS operates in different switching modes.

The inductive coupling approach and its application in EMI filter design have been extensively studied in the literature, but the variation characteristics of the in-circuit impedance have not been analyzed. Wang et al. modeled the switches as noise voltage or current sources to derive the equivalent EMI model for an SMPS and proposed EMI suppression techniques [18,19,20]. However, in these articles, the in-circuit impedance is derived based on the noise source substitution approach, revealing differences in the derived impedance under different substitution methods. Therefore, a novel method for the in-circuit impedance modeling is proposed for an SMPS, and its variation characteristics are elaborately analyzed. The main contributions of the paper can be concluded as follows:

(1) Based on the concept of the inductive coupling approach, a novel method for in-circuit impedance modeling is proposed.
(2) Based on the proposed method, the in-circuit impedance modeling for a buck converter and any SMPS with n switching modes is derived.
(3) Based on the derived model, the influencing factors of the in-circuit impedance for the SMPS are analyzed, and the variation characteristics are revealed.

The organization of this article is as follows: Section 2 analyzes the differences in in-circuit impedances after different substitution methods. In Section 3, a novel in-circuit impedance modeling method is proposed based on the concept of the inductive coupling approach, and an in-circuit impedance modeling for an SMPS is derived. In Section 4, the variation characteristics of in-circuit impedance for an SMPS are analyzed and verified by simulation. In Section 5, experiments are conducted to verify the proposed model. Section 6 concludes the article.

## 2. The Differences in In-Circuit Impedances After Different Substitution Methods

**Figure 1:**
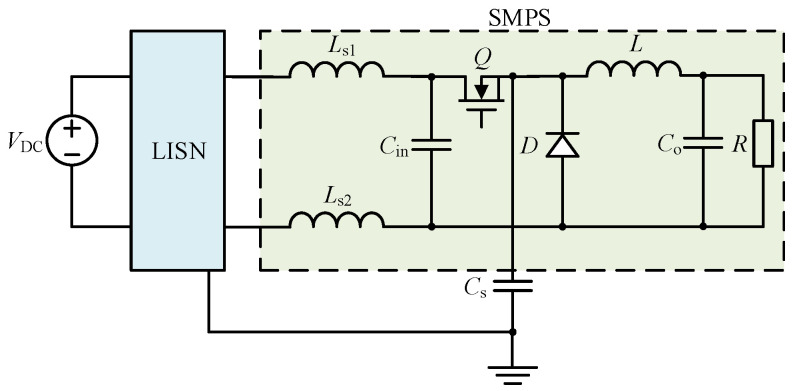
The buck converter selected to analyze the differences in in-circuit impedances after different substitution methods.

The noise source substitution approach is widely used to derive the equivalent EMI model for an SMPS. In Section 2, the DM impedance of the buck converter shown in Figure 1 is selected to analyze the differences in in-circuit impedances after different substitution methods. It is assumed that the buck converter operates in discontinuous conduction mode (DCM) with a switching period of *T*_s_. During one switching cycle, the conduction time of the switch *Q* is *D*_1_*T*_s_, and the conduction time of the diode *D* is *D*_2_*T*_s_, while the time during which both *Q* and *D* are OFF is *D*_3_*T*_s_, with *D*_1_ + *D*_2_ + *D*_3_ = 1. *V*_DC_ is the input DC voltage. *L*_s1_ and *L*_s2_ are the equivalent inductances of the input cables. *L* is the filter inductance. *C*_in_ is the input capacitor. *C*_o_ is the output capacitor. *C*_s_ is the parasitic capacitance between the source terminal of *Q* and the ground. *R* is the load, LISN is the line impedance stabilization network.

**Figure 2:**
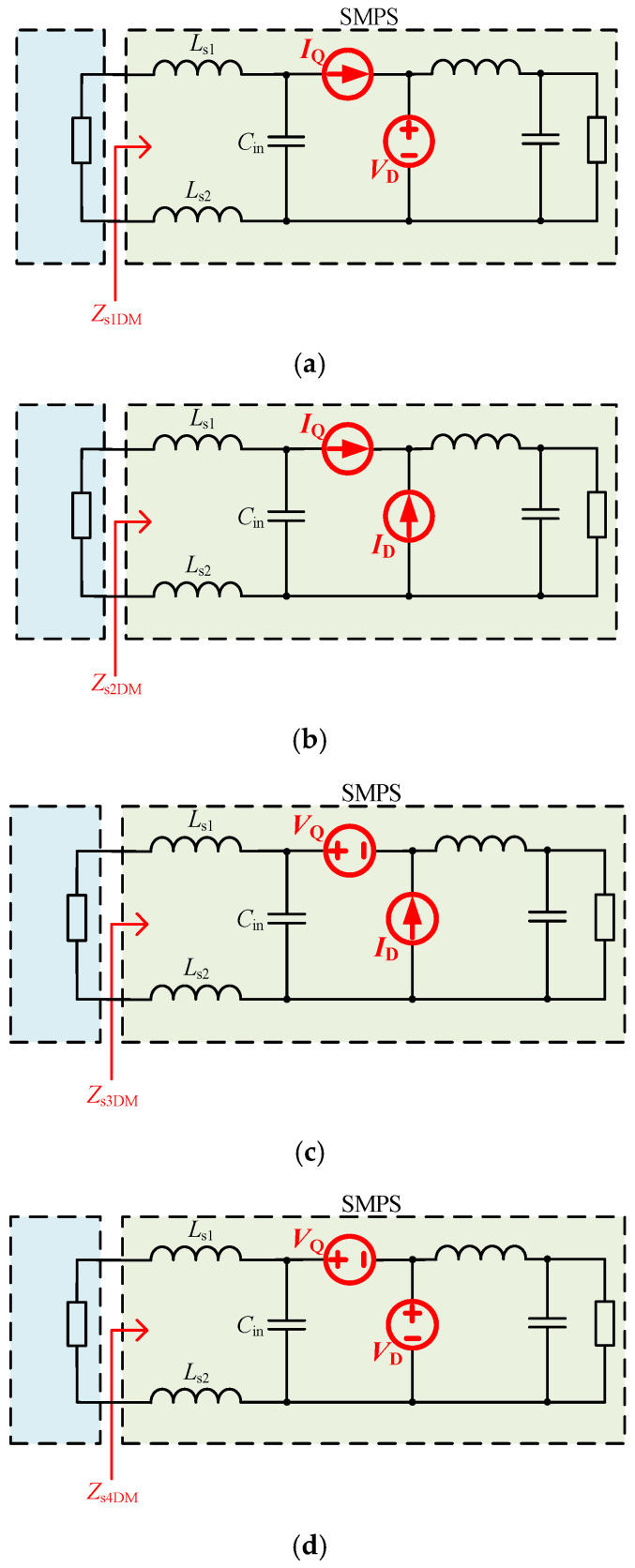
The equivalent DM circuits after four substitution methods. (**a**) Substitution method 1. (**b**) Substitution method 2. (**c**) Substitution method 3. (**d**) Substitution method 4.

The switch *Q* and diode *D* could be replaced by noise voltage sources or current sources, resulting in four possible substitution methods: (1) *Q* is replaced by a noise current source *I*_Q_, and *D* is replaced by a noise voltage source *V*_D_; (2) *Q* is replaced by a noise current source *I*_Q_, and *D* is replaced by a noise current source *I*_D_; (3) *Q* is replaced by a noise voltage source *V*_Q_, and *D* is replaced by a noise current source *I*_D_; and (4) *Q* is replaced by a noise voltage source *V*_Q_, and *D* is replaced by a noise voltage source *V*_D_. The equivalent differential-mode (DM) circuits after the four substitution methods are shown in Figure 2. *Z*_LISN_ is the impedance of LISN. It is assumed that *Z*_Ls1_, *Z*_Ls2_, *Z*_L_, *Z*_Cin_, *Z*_Co_, and *Z*_R_ are the impedances of *L*_s1_, *L*_s2_, *L*, *C*_in_, *C*_o_, and *R*. Based on Thevenin’s theorem, by short-circuiting the voltage sources and open-circuiting the current sources, the expressions for the DM impedances *Z*_s1DM_, *Z*_s2DM_, *Z*_s3DM_, and *Z*_s4DM_ after four substitution methods are given by

(1) $$Z_{s1\mathrm{DM}}=Z_{s2\mathrm{DM}}=Z_{\mathrm{Ls}1}+Z_{\mathrm{Ls}2}+Z_{\mathrm{Cin}}$$

(2) $$Z_{s3\mathrm{DM}}=Z_{\mathrm{Ls}1}+Z_{\mathrm{Ls}2}+Z_{\mathrm{Cin}}//\left(Z_{L}+Z_{\mathrm{Co}}//Z_{R}\right)$$

(3) $$Z_{s4\mathrm{DM}}=Z_{\mathrm{Ls}1}+Z_{\mathrm{Ls}2}$$

As shown in (1), (2), and (3), the in-circuit impedance varies when different noise source substitution methods are employed.

Therefore, the correct expression for the in-circuit impedance cannot be obtained by simply using the noise source substitution approach.

## 3. A Novel In-Circuit Impedance Modeling Method

To address the limitations of the traditional noise source substitution method, this paper proposes a novel in-circuit impedance modeling approach. This method is based on the general principle of weighted combination of switching modes and linearization, and its modeling process does not rely on specific voltage/current relationships of any particular topology. Therefore, as long as a system can be described by piecewise linear time-varying equations, the model is theoretically applicable to any switched-mode power supply topology with an arbitrary number of switching modes (e.g., boost, buck–boost, multilevel converters, etc.).

**Figure 3:**
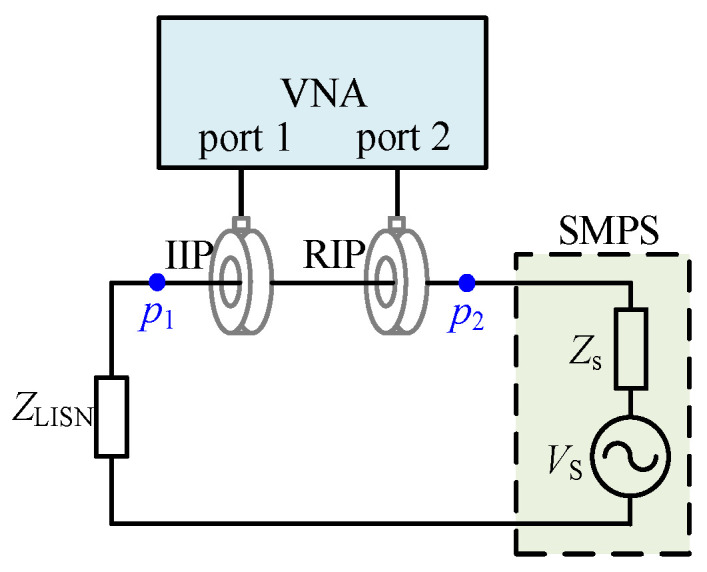
The schematic of the inductive coupling approach for the in-circuit impedance of an SMPS.

The schematic of the inductive coupling approach for the in-circuit impedance of an SMPS is shown in Figure 3. The VNA injects and receives signals through the IIP and RIP, respectively. Both ports of the VNA (port 1 and port 2) are set to 50 Ω impedance. The in-circuit impedance is calculated from the S-parameters measured by the VNA.

In addition, the single-probe setup based on the inductive coupling approach has also been extensively studied. Both approaches involve injecting a small signal into the circuit, using the VNA to receive the response signal and calculating the in-circuit impedance. Based on the above concept, by injecting an ideal small signal into the SMPS model, the in-circuit impedance can be calculated, thereby establishing an in-circuit model for the SMPS.

**Figure 4:**
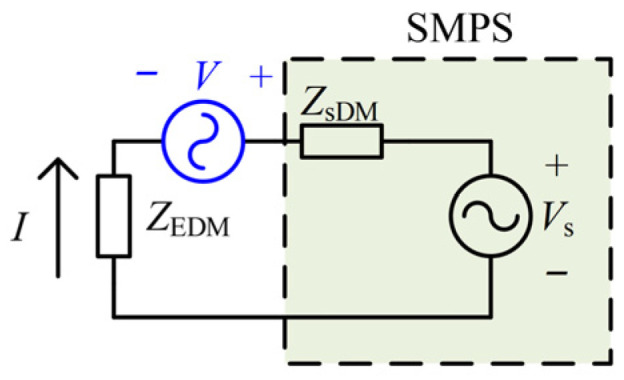
The equivalent DM circuit with the injected signal source in series.

Based on the concept of the inductive coupling approach, an ideal small-signal voltage source *v*(*t*) is connected in series in the circuit to simulate the injected signal, which is a sinusoidal wave with a frequency of *f* and an amplitude of *V*. The frequency *f* is equal to neither the switching frequency of the SMPS nor its multiples. The equivalent DM circuit with the injected signal source in series is shown in Figure 4. *Z*_sDM_ and *Z*_EDM_ represent the DM impedance and the external impedance of the SMPS, respectively, and *Z*_EDM_ is equal to *Z*_LISN_ in Figure 1. *V*_s_ represents the equivalent power noise voltage of the SMPS. *I* represents the equivalent current flowing through the external impedance *Z*_EDM_. At the frequency of *f*, *I* is equivalent to the current resulting from the sole effect of the injected signal source, which is given by

(4) $$I\left(f\right)=\frac{V\left(f\right)}{Z_{\mathrm{sDM}}\left(f\right)+Z_{\mathrm{EDM}}\left(f\right)}$$

Because the buck converter features a simple structure and clear switching modes, the modeling process of the equivalent noise source impedance is analyzed in detail based on the three switching modes of the power switch under the discontinuous conduction mode (DCM). Once the buck converter operates under DCM, it can be divided into three switching modes. Mode 1 corresponds to *Q* being on and *D* being off, Mode 2 corresponds to *D* being on and *Q* being off, while Mode 3 corresponds to both *D* and *Q* being off. The proportions of Mode 1, Mode 2, and Mode 3 are *D*_1_, *D*_2_, and *D*_3_, respectively.

**Figure 5:**
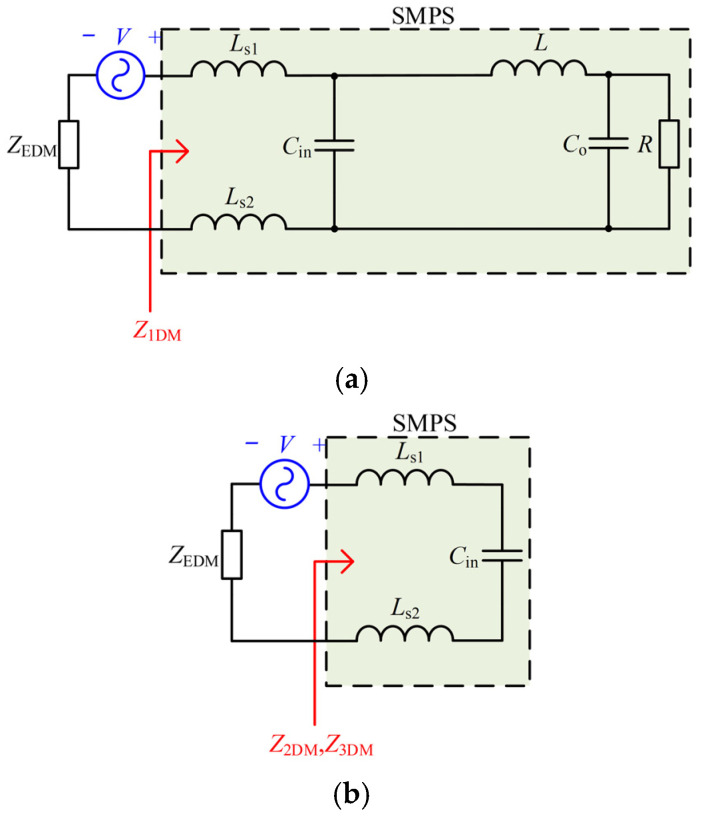
The equivalent DM circuits under Mode 1, Mode 2, and Mode 3 with the injected signal source in series. (**a**) Mode 1. (**b**) Mode 2 and Mode 3.

The modal impedances correspond to the input impedances of the Thevenin equivalent circuits under different switching modes. The equivalent DM circuits under Mode 1, Mode 2, and Mode 3 with the injected signal source in series are shown in Figure 5. The DM modal impedances under Mode 1, Mode 2, and Mode 3, denoted as *Z*_1DM_, *Z*_2DM_, and *Z*_3DM_, can be expressed as

(5) $$Z_{1\mathrm{DM}}=Z_{\mathrm{Ls}1}+Z_{\mathrm{Ls}2}+Z_{\mathrm{Cin}}//\left(Z_{L}+Z_{\mathrm{Co}}//Z_{R}\right)$$

(6) $$Z_{2\mathrm{DM}}=Z_{3\mathrm{DM}}=Z_{\mathrm{Ls}1}+Z_{\mathrm{Ls}2}+Z_{\mathrm{Cin}}$$

Assuming that the sampling function, denoted as *s*_1_(*t*), can be given by

(7) $$s_{1}\left(t\right)=\left\{\begin{array}{c} 1,nT_{s}\leq t<nT_{s}+D_{1}T_{s}\\ 0,nT_{s}+D_{1}T_{s}\leq t<\left(n+1\right)T_{s} \end{array}\right.,n=0,\pm 1,\pm 2,\ldots$$

when the buck converter operates in Mode 1, *s*_1_(*t*) = 1. As the impedances in Mode 2 and Mode 3 are identical, and assuming that the sampling function, denoted as *s*_2_(*t*), can be given by

(8) $$s_{2}\left(t\right)=1-s_{1}\left(t\right)$$

when the Buck converter operates in Mode 2 and Mode 3, *s*_2_(*t*) = 1.

The equivalent current flowing through *Z*_EDM_ in Mode 1, denoted as *i*_1_(t), can be expressed as follows:

(9) $$i_{1}\left(t\right)=s_{1}\left(t\right)\frac{v\left(t\right)}{Z_{\mathrm{EDM}}+Z_{1\mathrm{DM}}}$$

The equivalent current flowing through *Z*_EDM_ in Mode 2 and Mode 3, denoted as *i*_2_(t), can be expressed as follows:

(10) $$i_{2}\left(t\right)=s_{2}\left(t\right)\frac{v\left(t\right)}{Z_{\mathrm{EDM}}+Z_{2\mathrm{DM}}}$$

The equivalent current flowing through *Z*_EDM_, denoted as *i*(t), can be expressed as follows:

(11) $$i\left(t\right)=i_{1}\left(t\right)+i_{2}\left(t\right)$$

By using the convolution property in the frequency domain, (11) can be transformed as follows:

(12) $$\begin{array}{l} I\left(j\omega \right)=I_{1}\left(j\omega \right)+I_{2}\left(j\omega \right)\\ \;\;\;\;\;\;\;=\frac{1}{2\pi \left(Z_{\mathrm{EDM}}+Z_{1\mathrm{DM}}\right)}S_{1}\left(j\omega \right)⊗V\left(j\omega \right)\\ \;\;\;\;\;\;\;\;+\frac{1}{2\pi \left(Z_{\mathrm{EDM}}+Z_{2\mathrm{DM}}\right)}S_{2}\left(j\omega \right)⊗V\left(j\omega \right) \end{array}$$

Assuming that the fundamental angular frequency *ω*_s_ = 2π/*T*_s_, the Fourier coefficients of *s*_1_(*t*) can be expressed as

(13) $$\begin{array}{l} S_{1n}=\frac{1}{T_{s}}\int_{-\frac{T_{s}}{2}}^{\frac{T_{s}}{2}}s_{1}\left(t\right)e^{-jn\omega_{s}t}dt\\ \;\;\;\;\;\;=D_{1}\frac{\sin\left(\frac{n\omega_{s}D_{1}T_{s}}{2}\right)}{\frac{n\omega_{s}D_{1}T_{s}}{2}}=D_{1}\mathrm{Sa}\left(\frac{n\omega_{s}D_{1}T_{s}}{2}\right) \end{array}$$

Therefore, the frequency spectrum function of *s*_1_(*t*), denoted as *S*_1_(*jω*), can be expressed as

(14) $$S_{1}\left(j\omega \right)=2\pi D_{1}\sum_{n=-\infty }^{\infty }\mathrm{Sa}\left(\frac{n\omega_{s}D_{1}T_{s}}{2}\right)\delta \left(\omega -n\omega_{s}\right)$$

Based on (14), *I*_1_(*jω*) can be determined as follows:

(15) $$\begin{array}{l} I_{1}\left(j\omega \right)=\frac{2\pi D_{1}\sum_{n=-\infty }^{\infty }\mathrm{Sa}\left(\frac{n\omega_{s}D_{1}T_{s}}{2}\right)\delta \left(\omega -n\omega_{s}\right)⊗V\left(j\omega \right)}{2\pi \left(Z_{\mathrm{EDM}}+Z_{1\mathrm{DM}}\right)}\\ \;\;\;\;\;\;\;\;\;\;\;=\frac{D_{1}}{Z_{\mathrm{EDM}}+Z_{1\mathrm{DM}}}\cdot \sum_{n=-\infty }^{\infty }\mathrm{Sa}\left(\frac{n\omega_{s}D_{1}T_{s}}{2}\right)V\left[j\left(\omega -n\omega_{s}\right)\right] \end{array}$$

Because *V*[*j*(*ω* − *nω*_s_)] ≠ 0 only when *n* = 0, when *jω* equals *f*, (15) can be transformed as follows:

(16) $$I_{1}\left(f\right)=\frac{D_{1}V\left(f\right)}{Z_{\mathrm{EDM}}\left(f\right)+Z_{1\mathrm{DM}}\left(f\right)}$$

Similarly, *I*_2_(*f*) can be given by

(17) $$I_{2}\left(f\right)=\frac{D_{2}V\left(f\right)}{Z_{\mathrm{EDM}}\left(f\right)+Z_{2\mathrm{DM}}\left(f\right)}$$

Substituting (16) and (17) into (18), *I*(*f*) can be determined as follows:

(18) $$I\left(f\right)=\frac{D_{1}V\left(f\right)}{Z_{\mathrm{EDM}}\left(f\right)+Z_{1\mathrm{DM}}\left(f\right)}+\frac{D_{2}V\left(f\right)}{Z_{\mathrm{EDM}}\left(f\right)+Z_{2\mathrm{DM}}\left(f\right)}$$

Substituting (18) into (4), *Z*_sDM_(*f*) can be determined as follows:

(19) $$Z_{\mathrm{sDM}}\left(f\right)=\frac{Z_{\mathrm{EDM}}\left(f\right)+Z_{1\mathrm{DM}}\left(f\right)}{D_{1}}//\frac{Z_{\mathrm{EDM}}\left(f\right)+Z_{2\mathrm{DM}}\left(f\right)}{D_{2}}-Z_{\mathrm{EDM}}\left(f\right)$$

Extending (19) to the entire frequency range, *Z*_sDM_ of the buck converter can be determined as follows:

(20) $$Z_{\mathrm{sDM}}=\frac{Z_{\mathrm{EDM}}+Z_{1\mathrm{DM}}}{D_{1}}//\frac{Z_{\mathrm{EDM}}+Z_{2\mathrm{DM}}}{D_{2}}-Z_{\mathrm{EDM}}$$

Based on the above method for in-circuit impedance analysis, the in-circuit impedance modeling for any SMPS with n switching modes can be proposed. Assuming that the proportions of each switching mode are *D*_1_, *D*_2_, …, *D*_n_ and the DM modal impedances in each switching mode are *Z*_1DM_, *Z*_2DM_, …, *Z*_nDM_, *Z*_sDM_ can be given by

(21) $$Z_{\mathrm{sDM}}=\frac{Z_{\mathrm{EDM}}+Z_{1\mathrm{DM}}}{D_{1}}//\frac{Z_{\mathrm{EDM}}+Z_{2\mathrm{DM}}}{D_{2}}//\ldots //\frac{Z_{\mathrm{EDM}}+Z_{\mathrm{nDM}}}{D_{n}}-Z_{\mathrm{EDM}}$$

In existing studies, the impedance of the input capacitor is commonly treated as the DM impedance due to the noise source substitution approach. Although this simplification has little impact in the frequency range below 10 MHz, it can lead to significant inaccuracies in EMI modeling at higher frequencies.

In contrast, the model proposed in this section is not a single equivalent impedance. Instead, it is analytically expressed as a time-weighted combination of the corresponding impedances (modal impedances) under each switching mode over one switching cycle. This approach not only provides port characteristic prediction accuracy comparable to that of classical methods, but, more importantly, it directly relates the dynamics of online impedance to the physical process of switching modes. This enables quantitative analysis of the contribution of different switching states to the overall impedance. Thereby, by using the proposed in-circuit impedance modeling, the accuracy of the equivalent EMI model for the SMPS can be further improved, which is helpful for the optimization of EMI suppression techniques.

**Figure 6:**
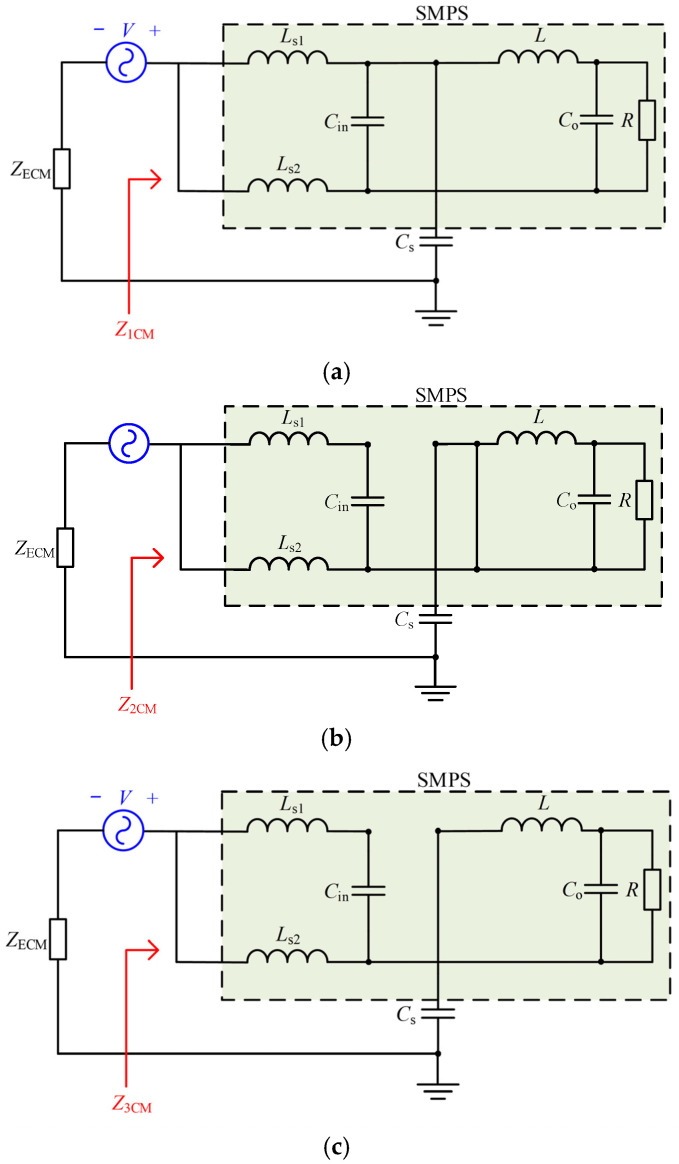
The equivalent CM circuits under Mode 1, Mode 2, and Mode 3 with the injected signal source in series. (**a**) Mode 1. (**b**) Mode 2. (**c**) Mode 3.

The equivalent CM circuits under Mode 1, Mode 2, and Mode 3 with the injected signal source in series are shown in Figure 6. It is assumed that *Z*_Cs_ is the impedance of *C*_s_. The CM modal impedances under Mode 1, Mode 2, and Mode 3, denoted as *Z*_1CM_, *Z*_2CM_, and *Z*_3CM_, can be expressed as

(22) $$Z_{1\mathrm{CM}}=Z_{\mathrm{Ls}1}//\left(Z_{\mathrm{Ls}2}+Z_{\mathrm{Cin}}//\left(Z_{L}+Z_{\mathrm{Co}}//Z_{R}\right)\right)+Z_{\mathrm{Cs}}$$

(23) $$Z_{2\mathrm{CM}}=Z_{\mathrm{Ls}2}//\left(Z_{\mathrm{Ls}1}+Z_{\mathrm{Cin}}\right)+Z_{\mathrm{Cs}}$$

(24) $$Z_{3\mathrm{CM}}=Z_{\mathrm{Ls}2}//\left(Z_{\mathrm{Ls}1}+Z_{\mathrm{Cin}}\right)+Z_{L}+Z_{\mathrm{Co}}//Z_{R}+Z_{\mathrm{Cs}}$$

*Z*_sCM_ of the buck converter can be determined as follows:

(25) $$Z_{\mathrm{sCM}}=\frac{Z_{\mathrm{ECM}}+Z_{1\mathrm{CM}}}{D_{1}}//\frac{Z_{\mathrm{ECM}}+Z_{2\mathrm{CM}}}{D_{2}}//\frac{Z_{\mathrm{ECM}}+Z_{3\mathrm{CM}}}{D_{3}}-Z_{\mathrm{ECM}}$$

Assuming that the proportions of each switching mode are *D*_1_, *D*_2_, …, *D*_n_ and that the CM modal impedances in each switching mode are *Z*_1CM_, *Z*_2CM_, …, *Z*_nCM_, *Z*_sCM_ can be given by

(26) $$Z_{s}=\frac{Z_{\mathrm{ECM}}+Z_{1\mathrm{CM}}}{D_{1}}//\frac{Z_{\mathrm{ECM}}+Z_{2\mathrm{CM}}}{D_{2}}//\ldots //\frac{Z_{\mathrm{ECM}}+Z_{\mathrm{nCM}}}{D_{n}}-Z_{\mathrm{ECM}}$$

## 4. Variation Characteristics Analysis of the In-Circuit Impedance for the SMPS

### 4.1. Variation Characteristics Analysis

As shown in (21) and (26), the in-circuit impedance for the SMPS is related to the external impedance, the modal impedance under different switching modes, and the proportion of each switching mode.

Taking an SMPS with two switching modes as an example, the in-circuit impedance *Z*_s_ can be simplified as

(27) $$Z_{s}=\frac{Z_{1}Z_{2}+\left(Z_{1}D_{1}+Z_{2}D_{2}\right)Z_{E}}{Z_{E}+Z_{1}D_{2}+Z_{2}D_{1}}$$

Substituting *D*_2_ = 1 − *D*_1_ into (27), *Z*_s_ can be expressed as follows:

(28) $$Z_{s}=\frac{\left(Z_{1}-Z_{2}\right)D_{1}Z_{E}+\left(Z_{E}+Z_{1}\right)Z_{2}}{Z_{E}+Z_{1}+\left(Z_{2}-Z_{1}\right)D_{1}}$$

Substituting *D*_1_ = 1 − *D*_2_ into (27), *Z*_s_ can be expressed as follows:

(29) $$Z_{s}=\frac{\left(Z_{2}-Z_{1}\right)D_{2}Z_{E}+\left(Z_{E}+Z_{2}\right)Z_{1}}{Z_{E}+Z_{2}+\left(Z_{1}-Z_{2}\right)D_{2}}$$

When |*Z*_E_| > |*Z*_1_|, |*Z*_E_| > |*Z*_2_|, |*Z*_E_| > |*Z*_1_*Z*_2_|, *Z*_s_ expressed in (27) can be approximated by

(30) $$Z_{s}\approx Z_{1}D_{1}+Z_{2}D_{2}$$

On the contrary, when |*Z*_E_| < |*Z*_1_|, |*Z*_E_| < |*Z*_2_|, *Z*_s_ can be approximated by

(31) $$Z_{s}\approx \frac{Z_{1}}{D_{1}}//\frac{Z_{2}}{D_{2}}$$

Based on (28)–(31), the following conclusions can be obtained:

(1) The greater *D*_1_ is, the longer the SMPS operates in Mode 1, and the closer *Z*_s_ approaches the modal impedance under Mode 1 *Z*_1_. Similarly, the greater *D*_2_ is, the longer the SMPS operates in Mode 2, and the closer *Z*_s_ approaches the modal impedance under Mode 2 *Z*_2_;
(2) When *Z*_E_ is much greater than *Z*_1_ and *Z*_2_, *Z*_s_ approaches *Z*_1_*D*_1_ + *Z*_2_*D*_2_; when *Z*_E_ is much smaller than *Z*_1_ and *Z*_2_, *Z*_s_ approaches (*Z*_1_/*D*_1_)//(*Z*_2_/*D*_2_).

The DM impedance of the buck converter operating in continuous conduction mode (CCM) shown in Figure 1 is selected for the following analysis. The parameters of the circuit are listed in Table 1. *ESL* is the series parasitic inductance of *C*_in_. *ESR* is the series parasitic resistance of *C*_in_. *EPC* is the parallel parasitic capacitance of *L*.

**Figure 7:**
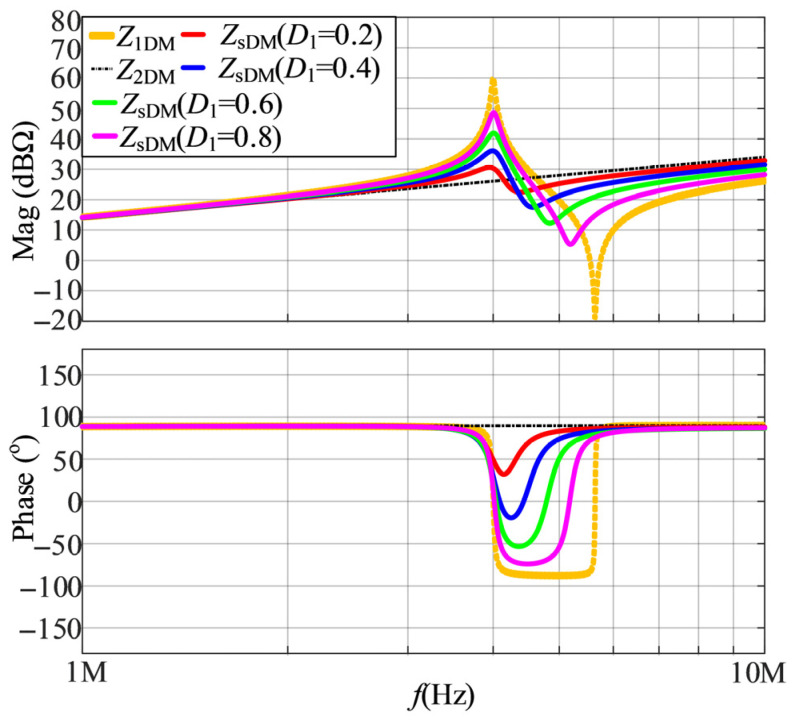
The calculated results of DM impedances when *Z*_EDM_ is equal to *Z*_LISN_ and the values of *D*_1_ are 0.2, 0.4, 0.6, and 0.8.

Based on (20), when *Z*_EDM_ is equal to *Z*_LISN_ and the values of *D*_1_ are 0.2, 0.4, 0.6, and 0.8, the calculated results of DM impedances are shown in Figure 7. It is shown that as *D*_1_ increases, the second resonant frequency of *Z*_sDM_ increases and the first resonant frequency remains nearly constant, while the phase variation of *Z*_sDM_ becomes more significant.

**Figure 8:**
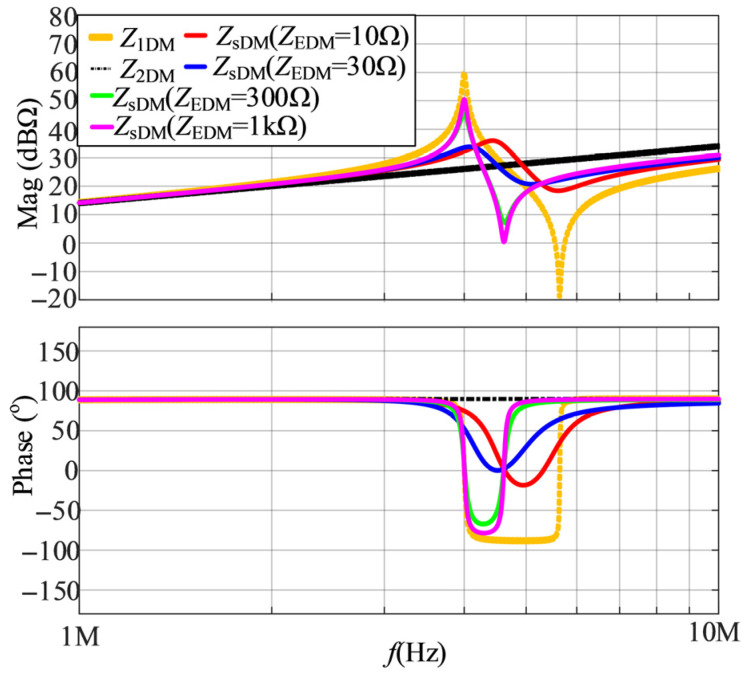
The calculated results of DM impedances when the value of *D*_1_ is 0.5 and *Z*_EDM_ are resistors of 10 Ω, 30 Ω, 300 Ω, and 1 kΩ.

When the value of *D*_1_ is 0.5 and *Z*_EDM_ are resistors of 10 Ω, 30 Ω, 300 Ω, and 1 kΩ, the calculated results of DM impedances are shown in Figure 8. It is shown that as *Z*_EDM_ increases, both of the two resonant frequencies of *Z*_sDM_ decrease, while the phase variation of *Z*_sDM_ first decreases and then increases.

Extending the above conclusions to any SMPS with n switching modes, the following conclusions can be obtained:

(1) The greater *D*_i_ (i = 1, 2, …, n) is, the closer *Z*_s_ approaches *Z*_i_ (i = 1, 2, …, n);
(2) When *Z*_E_ is much greater than *Z*_i_, *Z*_s_ approaches (32); when *Z*_E_ is much smaller than *Z*_i_, *Z*_s_ approaches (33).

(32) $$Z_{s}\approx \sum_{i=1}^{n}Z_{i}D_{i}$$

(33) $$Z_{s}\approx {\left(\sum_{i=1}^{n}\frac{D_{i}}{Z_{i}}\right)}^{-1}$$

### 4.2. Simulation Verification

To validate the above conclusions, the DM impedance of the buck converter shown in Figure 1 operating in CCM was selected for simulation verification. The inductive coupling approach was employed for simulation verification (13). Simulation parameters of the circuit are also listed in Table 1.

**Figure 9:**
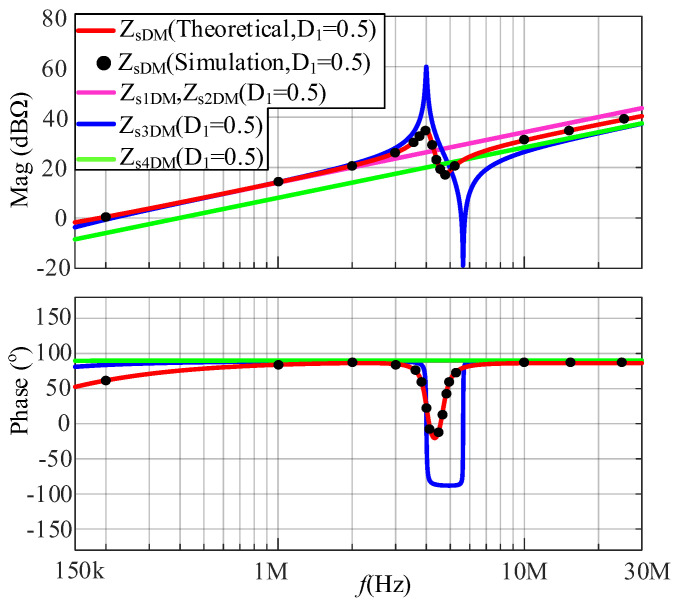
The comparison between the simulated and calculated results of DM impedances at a duty cycle of 0.5.

According to (20), the DM impedance at a duty cycle of 0.5 can be calculated. The comparison between the simulated and calculated results of the DM impedances is shown in Figure 9. It is shown that the calculated results are in good agreement with the simulated results, verifying the correctness of the proposed model. The DM impedances *Z*_s1DM_, *Z*_s2DM_, *Z*_s3DM_, and *Z*_s4DM_ after four substitution methods are also shown in Figure 9. Judging from Figure 9, the noise source substitution approach fails to derive the correct in-circuit impedance with large parasitic parameters.

**Figure 10:**
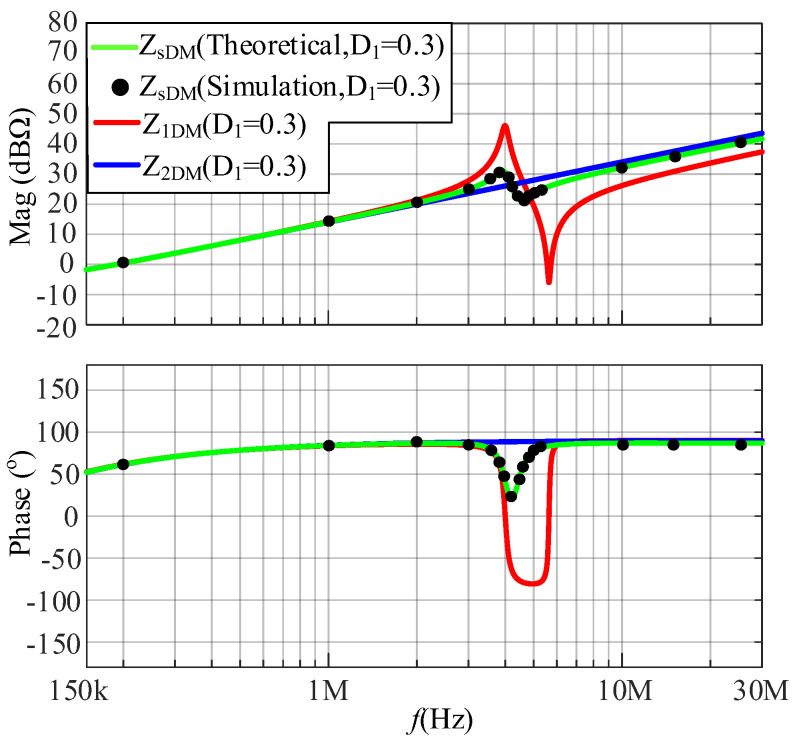
The comparison between the simulated and calculated results of DM impedances at a duty cycle of 0.3.

**Figure 11:**
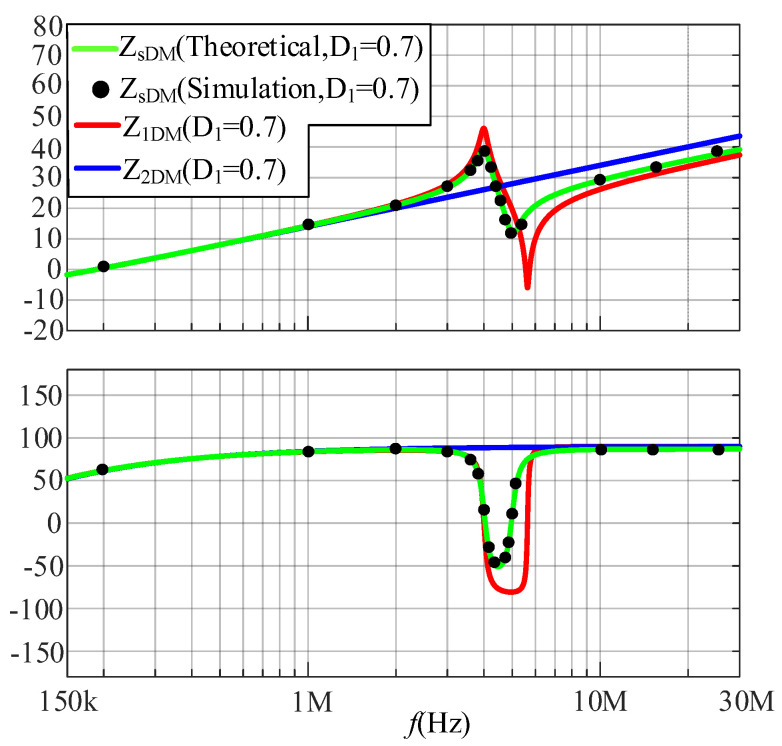
The comparison between the simulated and calculated results of DM impedances at a duty cycle of 0.7.

The comparisons between the simulated and calculated results of the DM impedance at a duty cycle of 0.3 and 0.7 are shown in Figure 10 and Figure 11, respectively. Based on Figure 10 and Figure 11, when *D*_1_ is 0.3 and *D*_2_ is 0.7, *Z*_sDM_ is closer to *Z*_2DM_; when *D*_1_ is 0.7 and *D*_2_ is 0.3, *Z*_sDM_ is closer to *Z*_1DM_. The following conclusion is verified: the greater *D*_i_ is, the closer *Z*_s_ approaches *Z*_i_.

**Figure 12:**
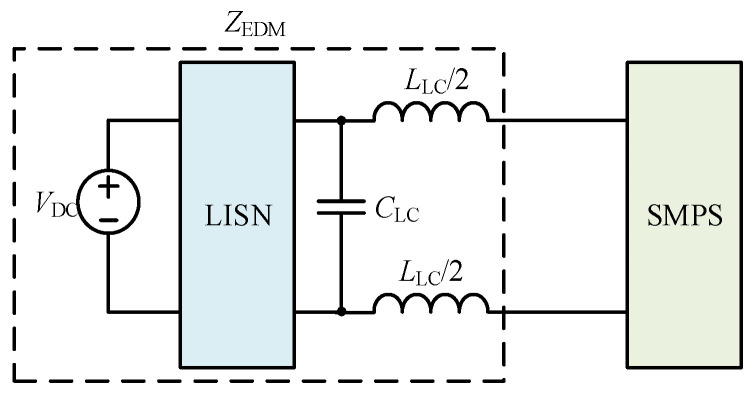
The schematic diagram after the insertion of the LC DM EMI filter.

**Figure 13:**
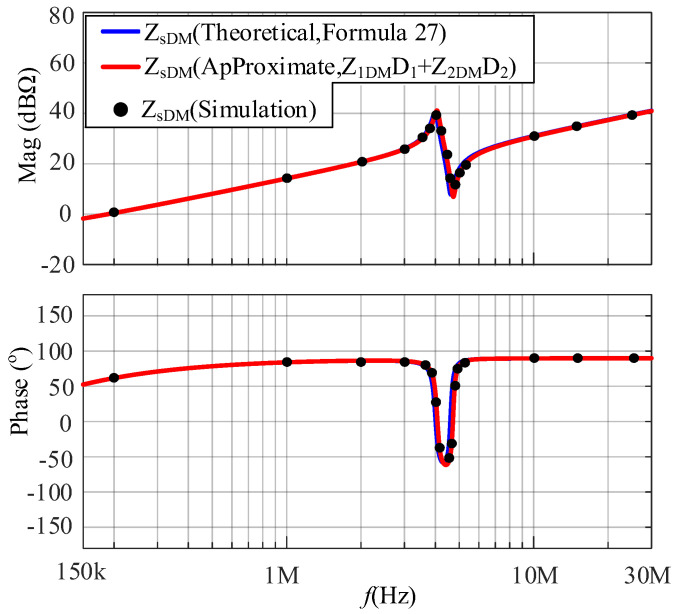
The comparison between the simulated and calculated results of DM impedances after inserting an LC DM EMI filter.

An LC DM EMI filter is inserted between the SMPS and the LISN as shown in Figure 12. The LC DM EMI filter is part of the *Z*_EDM_, with *L*_LC_ and *C*_LC_ values of 5 μH and 100 nF, respectively. In the frequency range of interest, *Z*_EDM_ is much greater than *Z*_1DM_ and *Z*_2DM_. The comparison between the simulated and calculated results of the DM impedance at a duty cycle of 0.5 after inserting an LC DM EMI filter is shown in Figure 13. Based on Figure 13, *Z*_s_ approaches *Z*_1_*D*_1_ + *Z*_2_*D*_2_.

**Figure 14:**
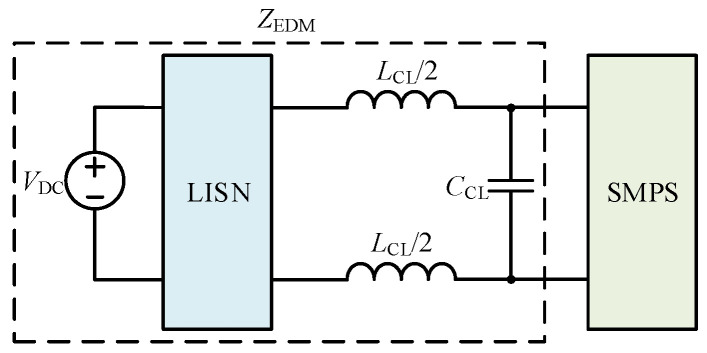
The schematic diagram after the insertion of the CL DM EMI filter.

**Figure 15:**
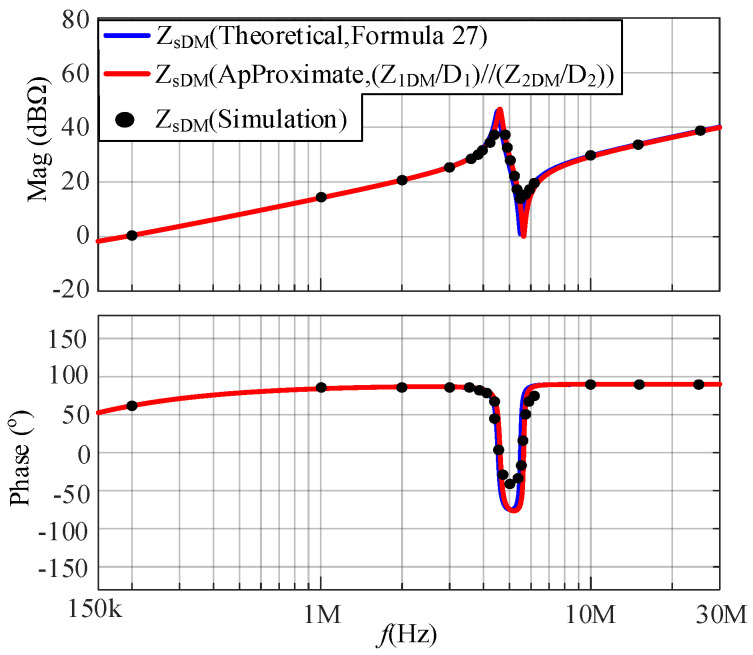
The comparison between the simulated and calculated results of DM impedances after inserting a CL DM EMI filter.

A CL DM EMI filter is inserted between the SMPS and the LISN as shown in Figure 14. The CL DM EMI filter is part of *Z*_EDM_, with *L*_CL_ and *C*_CL_ values of 100 μH and 100 nF, respectively. In the frequency range of concern, *Z*_EDM_ is much smaller than *Z*_1DM_ and *Z*_2DM_. The comparison between the simulated and calculated results of the DM impedance at a duty cycle of 0.5 after inserting a CL DM EMI filter is shown in Figure 15. Based on Figure 15, *Z*_s_ approaches (*Z*_1_/*D*_1_)//(*Z*_2_/*D*_2_).

From Figure 13 and Figure 15, the following conclusion is verified: when *Z*_E_ is much greater than *Z*_i_, *Z*_s_ approaches (32); when *Z*_E_ is much smaller than *Z*_i_, *Z*_s_ approaches (33).

## 5. Experimental Verification

To verify the correctness of the proposed model, a buck converter prototype operating in CCM was fabricated with the following specifications: input voltage *U*_in_ = 48 V, output power *P*_out_ = 400 W, and switching frequency *f*_sw_ = 200 kHz.

In this article, the inductive coupling approach is employed to extract the DM impedance of the experimental prototype (13). An R&S ZLN3 vector network analyzer, two MYCP01 current probes, and a 3Ctest LISN J50 are employed.

**Figure 16:**
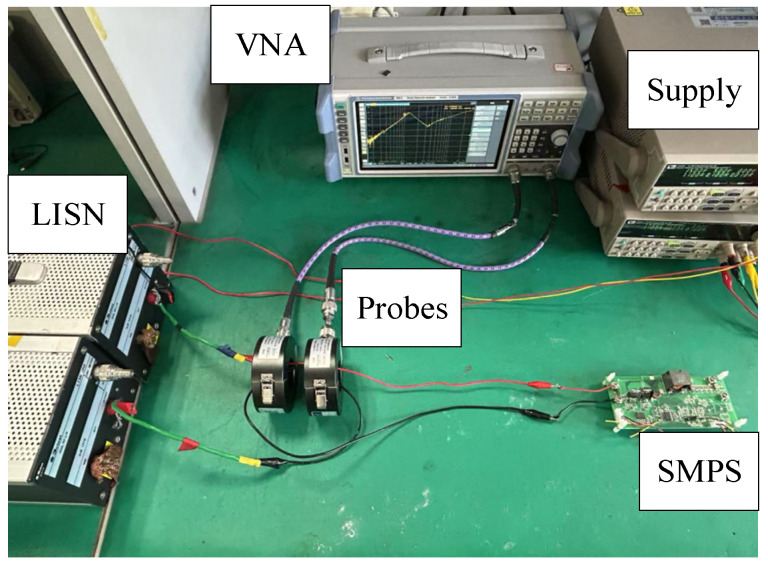
The DM impedance measurement setup for the SMPS.

**Figure 17:**
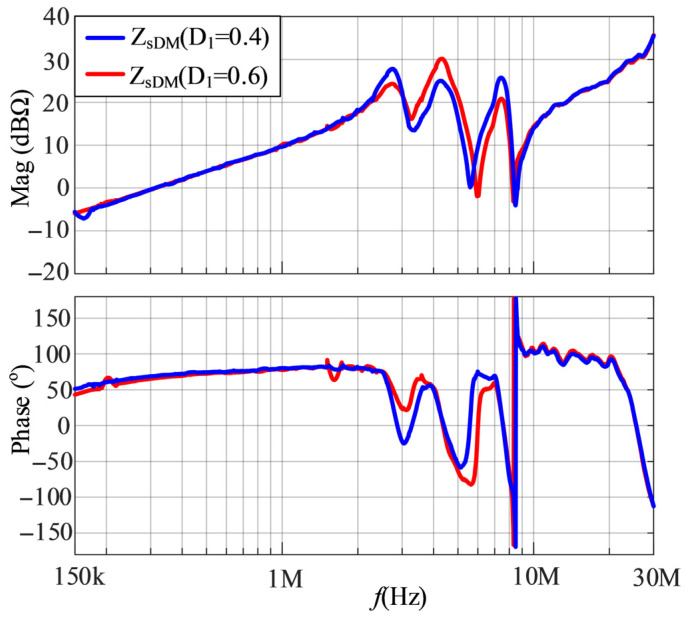
The DM impedances of the SMPS with duty cycles of 0.4 and 0.6.

The in-circuit impedance measurement setup for the SMPS is shown in Figure 16. Due to the parasitic parameters of the switch and diode and the DC bias characteristics of the capacitors and inductors, *Z*_1DM_ and *Z*_2DM_ cannot be directly measured. Therefore, firstly, DM impedances of the SMPS with duty cycles of 0.4 and 0.6 are obtained by the inductive coupling approach, as shown in Figure 17. From Figure 17, it is shown that the in-circuit impedances exhibit certain differences under different duty cycles.

**Figure 18:**
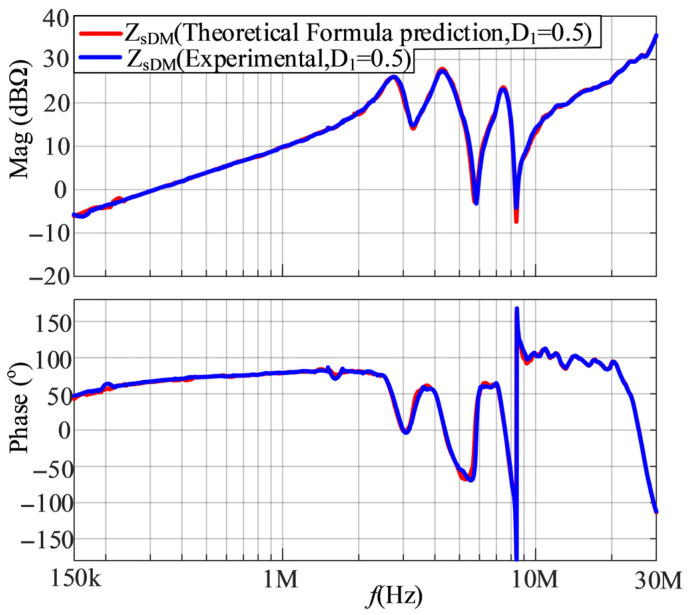
The comparison between the experimental and calculated results of DM impedances at a duty cycle of 0.5.

Given the DM impedances of the SMPS at duty cycles of 0.4 and 0.6, *Z*_1DM_ and *Z*_2DM_ can be derived by (20). Substituting *Z*_1DM_ and *Z*_2DM_ back into (20), the DM impedance of the SMPS at a duty cycle of 0.5 can be calculated. The comparison between the experimental and calculated results of DM impedances at a duty cycle of 0.5 is shown in Figure 18. Based on Figure 18, the calculated results are in good agreement with the experimental results, verifying the correctness of the proposed model.

## 6. Conclusions

In order to explore the variation characteristics of the in-circuit impedance for an SMPS, a novel method for in-circuit impedance modeling is proposed in this article based on the concept of the inductive coupling approach. Through an in-depth analysis of this model, the primary conclusions obtained are as follows:

(1) The in-circuit impedance of an SMPS is related to the external impedance, the modal impedance under different switching modes, and the proportion of each switching mode;
(2) The greater *D*_i_ is, the closer *Z*_s_ approaches *Z*_i_;
(3) When *Z*_E_ is much greater than *Z*_i_, *Z*_s_ approaches (32), and when *Z*_E_ is much smaller than *Z*_i_, *Z*_s_ approaches (33).

Through simulation and experimental verification, it is demonstrated that the proposed model can accurately characterize the in-circuit impedance of an SMPS, which can provide valuable guidance for the design of EMI suppression measures.

## Figures and Tables

**Table 1:** The Parameters of the circuit.

Parameter	Quantity
*L*_s1_/*L*_s2_ (μH)	0.2
*L* (μH)	37.5
*EPC* (nF)	4
*C*_in_ (μF)	10
*ESL* (nH)	400
*ESR* (mΩ)	100
*C*_o_ (μF)	100
*R* (Ω)	160

## Data Availability

The datasets generated and analyzed during this study are not currently publicly available as they form part of an on-going research project and are planned for use in forthcoming publications.
